# A Novel Transmittance Vis–NIR Hyper-Spectral Imaging Scanner for Analysis of Photographic Negatives: A Potential Tool for Photography Conservation

**DOI:** 10.3390/s23073562

**Published:** 2023-03-29

**Authors:** Costanza Cucci, Andrea Casini, Lorenzo Stefani, Barbara Cattaneo, Marcello Picollo

**Affiliations:** 1 Institute of Applied Physics “Nello Carrara” of the Consiglio Nazionale delle Ricerche (IFAC-CNR), Via Madonna del Piano, 10, 50019 Firenze, Italy; 2 Paper and Parchment Department of the Opificio delle Pietre Dure (OPD), Ministry of Culture, Viale F. Strozzi, 1, 50129 Firenze, Italy

**Keywords:** transmittance hyperspectral imaging, photographic negatives, photographic heritage conservation, Vis–NIR spectroscopy, non-invasive analysis, imaging spectroscopy

## Abstract

This work illustrates a novel prototype of a transmittance hyperspectral imaging (HSI) scanner, operating in the 400–900 nm range, and designed on purpose for non-invasive analysis of photographic materials, such as negatives, films and slides. The instrument provides high-quality spectral data and high-definition spectral images on targets of small size (e.g., 35 mm film strips) and is the first example of HSI instrumentation specifically designed for applications in the photographic conservation field. The instrument was tested in laboratory and on a set of specimens selected from a damaged photographic archive. This experimentation, though preliminary, demonstrated the soundness of a technical approach based on HSI for large-scale spectroscopic characterization of photographic archival materials. The obtained results encourage the continuation of experimentation of HSI as an advanced tool for photography conservation.

## 1. Motivation and Research Aim

This work illustrates a novel prototype of a transmittance hyperspectral imaging (HSI) scanner, operating in the 400–900 nm range, and specifically tailored for the non-invasive analysis of photographic negatives, films and slides. The instrument provides high-quality spectral data and high-definition spectral images and is the first example of HSI instrumentation specifically designed for applications in the photographic conservation sector. The interest in investigating the potentialities of the HSI technique for this niche applicative area is motivated by the growth in the use of analytical methods for identifying historical materials and technical processes in photographic heritage [1,2]. Among the several subtopics, the study and preservation of color photographic products are even more challenging due to the patents covering the industrial formulations of historical photographic brands.

Since, in other contexts, Visible and Near-Infrared (VNIR, 400–1000 nm) as well as Short-Wave Infra-Red (SWIR, 1000–2500 nm) spectroscopic methods have been successfully used for the non-invasive identification of pigments and dyes in colored plastics and polymeric materials [3,4,5], VNIR-SWIR spectroscopy is expected to be highly advantageous for the investigation of chromogenic processes, dyes detection and color degradation in color photographic products. 

In recent studies, spectroscopic analysis in the 400–2500 nm range was proposed, in combination with statistical analysis, for discriminating photographic paper manufacturers based on their fingerprints [6], or for dating gelatin silver photographs [7]. In addition, promising results were obtained with Ultraviolet (UV) and Visible (Vis) spectrophotometry to monitor the fading of dyes in colored slides [8]; in [9], dyes in Polaroid films were characterized by Fiber Optics Reflectance Spectroscopy (FORS) in the 400–1000 nm range. Thus, this state-of-the-art in the field paves the way for testing imaging spectroscopy, namely, HSI in the VNIR-SWIR range, as a new analytical technique for the non-invasive analysis of color photographic materials. Indeed, until today a lack of dedicated HSI instrumentation suitable for photographic specimens has inhibited the application of imaging spectroscopy in photographic heritage conservation. 

This paper illustrates the technical design and specifications of the transmittance HSI scanner prototype, which has been designed on purpose for the investigation of small-size photographic items, such as negatives, slides and films. The optical design guarantees accurate spatial and spectral sampling of these targets. The instrument was tested in the laboratory and on a set of specimens selected from a damaged photographic archive. This experimentation, though preliminary, demonstrated the soundness of a technical approach based on HSI for the large-scale spectroscopic characterization of photographic archival materials. The obtained results encourage the continuation of experimentation of HSI as an advanced tool for photography conservation.

## 2. Introduction: Hyperspectral Imaging Lacks in Photography Conservation

After about twenty years of dedicated applications in the field of Cultural Heritage, HSI is today acknowledged as a mature technology for the non-invasive analysis of several types of artworks and historical objects. The strength point of HSI techniques lies in the implementation of spectroscopic measurement principles (e.g., reflectance, transmittance, fluorescence, etc. modes) as an imaging acquisition mode. In this way, HSI provides a spectrum (in reflectance, transmittance, fluorescence, etc.) per each pixel of the imaged surface, whose color image is also provided (e.g., in RGB format) to visualize the target area. Thus, the resulting HSI data set, called ‘data cube’ (or ‘image cube’), consists of a collection of spectra tiling the target area. At the same time, the HSI data set can be organized as a sequence of spectral images of the same area, acquired on subsequent, contiguous and narrow bands, covering an extended spectral region, e.g., the VNIR and/or the SWIR ranges. The extension of the spectral interval depends on the type of sensors (e.g., Si CCD or Si CMOS, InGaAs, MCT, etc.). By associating compositional information (spectrum) to each spatial point (pixel) of an image, the HSI data embed incredibly dense informative content about the target surface and its constituent materials [10]. This approach has proven to be especially useful for the non-invasive analysis of polychrome surfaces in artworks and heritage objects. In particular, since VNIR-SWIR reflectance spectroscopy is especially effective for the identification of pigments, dyes and some alteration products, as well as for the colorimetric analysis, HSI in reflectance-mode has been widely applied to different artworks, such as paintings [11,12], illuminated manuscripts [13], books and graphical documents [14], and, more recently, also to mural paintings, frescoes and archaeological assets [15]. However, to the best of the authors’ knowledge, until todayHSI has not yet been applied in the field of photographic conservation, essentially because of a lack of dedicated instrumentation featuring the technical characteristics for investigating photographic materials. 

Although they are not yet fully recognized as part of the traditional cultural heritage, photo and film materials—gathered in public and private archives and collections, or even available as single erratic items—are invaluable repositories of our culture and recent history. On the other hand, photography and film are arts of the modern eras: thus, full awareness of their transience has been reached only recently, along with the increasing concerns on their rapid and massive degradation [16,17]. The photographic heritage is huge and highly varied, and its preservation is challenging. A necessary prerequisite is the knowledge of the materials and chemical products employed in producing the final products (photos, films, slides, etc.), as well as the production processes [18]. The peculiarity of photo-sensitive materials, the diversity of photographic supports, and the complexity of chemical processes involved in degradation processes pose very hard challenges to set up suitable conservation strategies [19,20,21]. In this perspective, building databases of reference materials (e.g., dyes, supports, etc.) appears as a necessary step to set up sound experimentations for both restoration and preventive conservation. However, the task of classifying the basic materials and constituents in photographic collections is particularly difficult, because photographic products are yields of industrial manufacturing, and hence can be covered by patents, or subject to changes over the years, following the upgrades of formulations and production technologies. The problem of cataloging the constitutive materials becomes even more challenging with color photographic products (printed photographs, films and negatives), which are more recent, and then more subject to brand policies, but, notwithstanding, highly exposed to degradation risks. [22]. Indeed, the constitutive materials (supports, dyes, emulsions, etc.) of chromogenic photography are extremely prone to fading and chromatic losses, and thus methods for their preservation and restoration are highly investigated [23,24,25,26,27].

The present work focuses on the topic of non-invasive analysis for the preservation and rescue of color negatives. Several approaches are taken today to recover image losses in color negatives. Besides the more conventional methods based on the photochemical restoration of the original photographic negatives, a very promising way to cope with the loss of content in color negatives is envisaged in transferring the visual contents to other supports, by means of different technologies, such as re-printing on a polyester base, or by using digital reproduction [26,28,29,30,31,32]. These methods can offer interesting advantages in terms of digital recovery of colors, digital restoration and reconstruction of the original image. This approach builds on the knowledge of the original materials (polymeric bases, emulsions, dyes, etc.), which is attained using spectroscopic methods [33,34,35]. In general, to obtain a complete identification of the materials and gain insights into the used photographic processes, a multi-analytical approach might be needed, based on optical, vibrational and X-ray spectroscopies [36,37,38]. Among the possible techniques, optical UV-VNIR spectroscopy implemented as FORS has successfully been used in photography conservation for the fast non-invasive characterization of dyes, as well as for color characterization [6,7,8,9]. Thus, the use of imaging spectroscopy appears as a natural subsequent step forward. Up to now, the pioneering use of multispectral imaging (MSI) technology for an accurate color reproduction of films and negatives has provided promising results [39,40]. With respect to MSI, HSI could offer the additional advantage of spectral characterization, meaning that compositional information is attainable using high-resolution spectroscopy. In addition, the application of chemometric methods to HSI data processing appears highly promising for classifying materials in massive data sets, as is the case for photographic archives and collections [6]. Despite these advantages, HSI still remains disregarded for these applications. The main reason is the technical inadequacy of existing devices for operating on these specific materials. Indeed, the HSI instrumentation used in cultural heritage typically operates in reflectance mode, which is inappropriate for use on transparent and glossy supports, such as photographic negatives. Up to now, the use of HSI imagers operating in transmittance for Cultural Heritage applications has been very sporadic [41], and, to the best authors’ knowledge, the unique HSI instrument designed on-purpose for photographic negatives was the prototype developed within the Project “Photographic Memory, Story of a collective recovery”, closed in 2019 with a final exposition and workshop [42]. This Project, primarily aimed at defining the guidelines and the technical protocols to rescue damaged photographic archives, also provided the opportunity to design the first available prototype of a high-performance transmittance HSI scanner tailored for applications on photographic negatives. The present paper illustrates the technical details and performances of this instrumental prototype, presenting a discussion of its applicative perspectives.

## 3. Experimental Section: The Transmittance HSI Scanner

### 3.1. Design

The HSI scanner for photographic negatives was designed at the IFAC-CNR laboratories as a variant of the existing reflectance HSI scanner prototype originally addressing the investigations of paintings [43,44,45]. The present release was instead readapted for transparent small-size targets, such as 35 mm negatives films.

**Figure 1:**
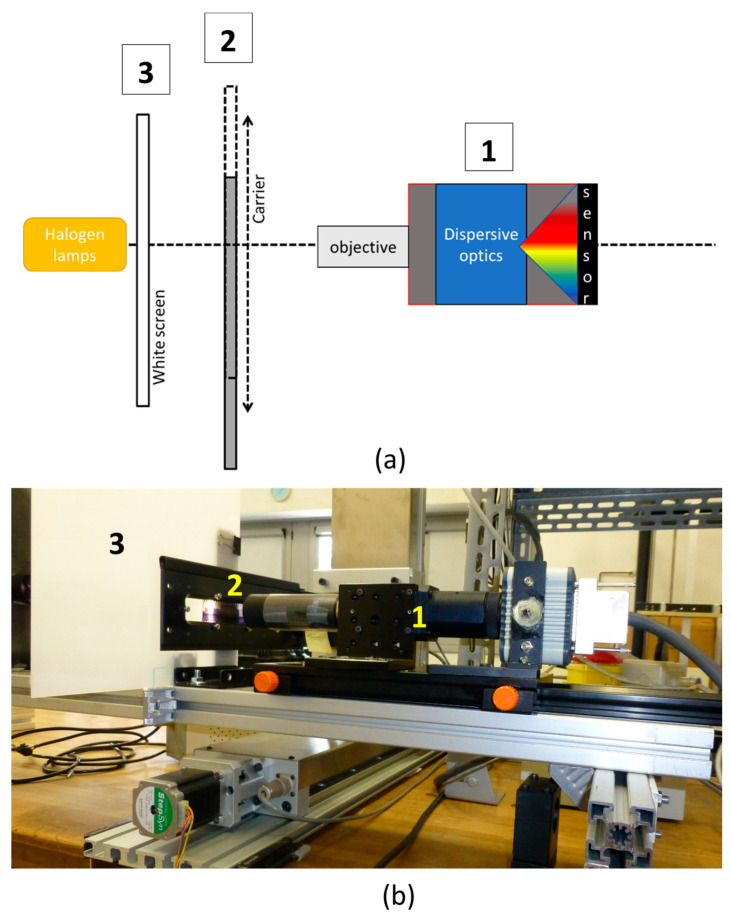
(**a**) Scheme of the setup and modules of the HSI transmittance scanner: the optical-spectrographic head (1), the automated scan strip carrier (2) and the screen of the illumination stage (3); (**b**) The three modules in (**a**) view of the prototype of the Transmittance IFAC-CNR HSI Scanner.

Therefore, the measurement mode was changed to transmittance, and the optical design was optimized to enable the analysis of sub-millimetric details. As sketched in Figure 1, the measurement set-up scheme includes three modules: the optical-spectrographic head, the automated scan strip carrier and the illumination stage. These blocks are lined up on a mechanical horizontal track enabling the adjustment of their mutual distances. The optical-spectrographic head consists of a push-broom HSI system (Specim^®^ mod. ImSpector N10E) coupled with a high-sensitivity camera (mod. ORCA-ERG Hamamatsu^®^) based on Silicon CCD 1344 × 1024 pixels detector. The camera head is equipped with a bi-telecentric objective (Opto-Engineering TL series), with a working distance of 67.2 mm, field depth of 5 mm and f/8 aperture. The use of telecentric optic guarantees the correction of possible geometrical distortions, possibly due to corrugated surfaces of deteriorated targets.

The adopted configuration is intended to fully frame the vertical dimension of a standard 35 mm negative, so that a negative strip can be imaged with sole one-direction scanning. The acquisition scan is performed by moving the target with respect to the camera. A strip carrier, placed at a fixed working distance from the objective, is mounted on an automated precision movement stage, sliding in the direction orthogonal to the optical axis. The streak covers 25 cm, enabling the acquisition of six standard photograms 24 × 36 mm at once. 

The illumination module is a standard slide projector, including a 150-Watt tungsten-quartz halogen QTH lamp with 3200 K color temperature, aligned with optical axis and coupled with a light-diffuser white screen, which creates a uniform illumination field on the target. The white screen is a plate made of polymeric material (PMA) designed for use in standard RGB scanners. The projector is equipped with a fan for heating dissipation. Although no significant temperature rise was detected on the screen during the laboratory tests, a filter holder inside the projector module enables the optional insertion of a heat reflective filter (ZSC1100 Super Cold Filter Asahi Spectra, Torrance, CA 90501 USA), in order to safely operate on highly sensitive targets. During the measurement, only the area to be acquired is illuminated, while the rest of the target is masked by a black cover, thus minimizing the overall light exposure of the target. With the scan speed being 1.67 mm/s, in the present configuration a single frame is acquired in about 1 min. The illuminance value measured on the target surface is about 1000 lux, and it is reduced to 180 lux when the filter is on.

The HSI system operates in the 400–900 nm spectral range with a spectral resolution of 2.8 nm. The spatial sampling step along the scan direction is approximately 0.4 mm (37 μm), providing a spatial sampling of about 27 points per millimeter in the acquired images. The corresponding spatial resolution in the spectral images is assessed as being equivalent to 700 ppi. 

The transmittance data are calculated upon white calibration and dark correction. The ‘white-reference’ calibration was performed ‘in-air’, by using as a reference the signal acquired on the white diffuser screen. The dark current noise was estimated by acquiring the average value of 100 dark images before each measurement session, and this value was subtracted from the measured data. 

### 3.2. Laboratory Tests 

**Figure 2:**
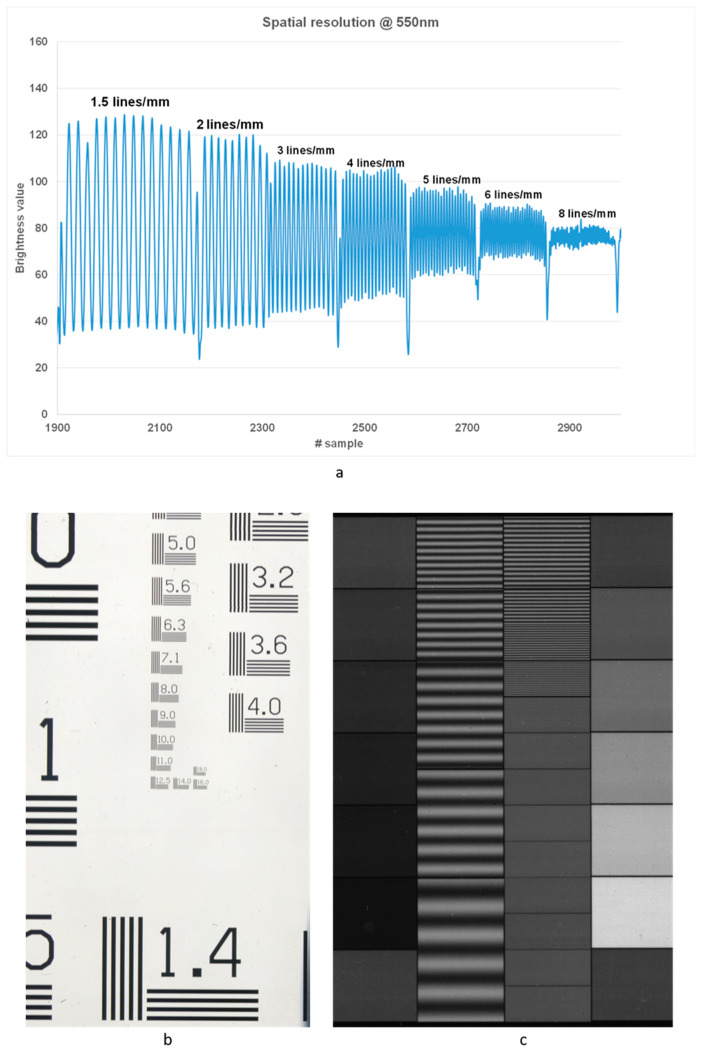
(**a**) Plot of contrast function in the image of the sinusoidal pattern HR target acquired at 550 nm. (**b**) The image acquired at 550 nm of the 1951 USAF target; (**c**) Image at 550 nm of the HR sinusoidal target.

The targeted application on photographic negatives requires high spatial resolving power; therefore, the overall spatial resolution of the system was tested by evaluating its MTF (Modular Transfer Function). Two different optical targets were used, namely: an optical sinusoidal target (Edmund Optics) and a 1951 USAF resolution test chart. The sharpness of profiles in the different spectral images was evaluated by adopting the 50% contrast resolution criterion (Figure 2). For example, in Figure 2a, the line-profile modulation pattern obtained from the spectral image of the USAF 1951 target (Figure 2b) acquired at 550 nm, is reported. As can be noticed, 50% of the peak contrast spatial is still guaranteed at the spatial frequency of 6 line pairs/mm. This value was confirmed by the acquisition of the sinusoidal target (Figure 2c). Although this value for the spatial resolution is below those typically provided by professional scanners used for digitization, it is, anyway, sufficient for ensuring a correct reproduction of the finest details in 35 mm negatives. Indeed, the attained resolution enables the extraction of spectroscopic information (transmittance spectrum) from single details of the scene. In principle, this makes possible the digital reconstruction of the lost colors of fine details based on their spectrum.

### 3.3. Applications to Photographic Materials: A Case Study within a Research Project 

**Figure 3:**
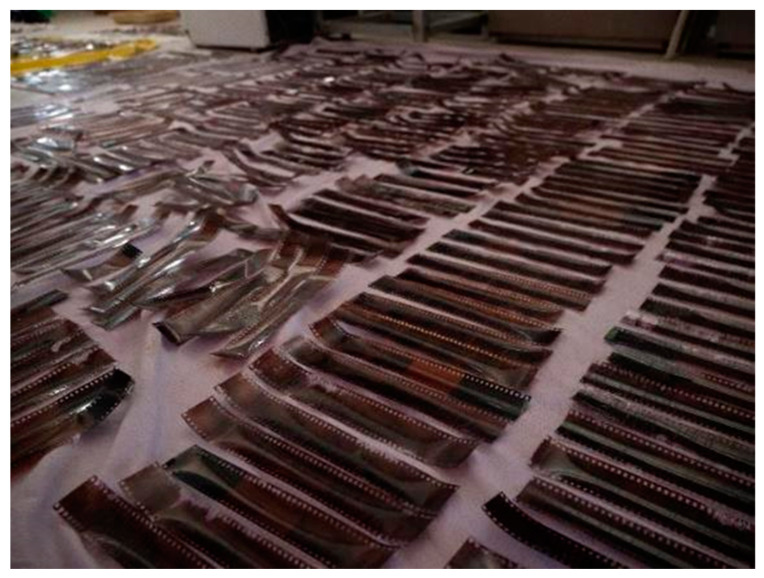
A view of the massive survey and recovering campaign of the Dainelli photographic archive.

After the earlier phase of laboratory tests, the effectiveness and the feasibility of using the HSI transmittance IFAC-CNR prototype for the analysis of photographic materials were fully demonstrated in the framework of the “Memoria Fotografica” research project, whose results are published in [42]. This Project was dedicated to the rescue of a contemporary photographic collection, the ‘Dainelli’ archive, which was flooded and seriously damaged in September 2017, when an extreme meteorological event struck the city of Livorno, in Tuscany (Italy) [42]. The archive, owned by the photographer Daniele Dainelli, included a highly representative number of photographic materials and supports in use in the late 20th Century (1980–1990). The collection comprised 7800 different items, such as color and B&W negatives, reversal films, color slides, B&W and color prints and ink-jet prints. Such a richness of contemporary photographic materials, collected over a narrow period of time (two decades), made this collection a valuable segment of the local historical photographic heritage. Being acknowledged as a high-value photographic collection, the local authorities (Tuscany Region, Italy) financed not just an action for rescuing the damaged archive, but a wider research project dedicated to heritage photography and related technological advances. The ultimate goal was, therefore, the development of an intervention protocol based on both traditional and innovative technologies. Thus, within the Project, after an earlier phase devoted to the prompt intervention on a massive number of flooded materials (Figure 3), a survey and diagnostics program was initiated, aimed at setting up the conservative priorities based on the data analysis. In this phase, well-established analytical techniques—such as Fourier Transform Infrared Spectroscopy (FT-IR) with Attenuated Total Reflectance (ATR) accessory, Scanning Electron Microscopy (SEM), and Optical Microscopy (OM)—were used to identify the main constituent materials and classify the main types of damage. Instead, as a novel technology for the photographic conservation field, the transmittance HSI scanner was proposed and tested in-field with preliminary measurements on selected negatives as case studies. Nevertheless, the lack of reference photographic materials mimicking the spectral behaviors of the original undamaged samples prevented the use of the acquired HSI data to attain analytical information on the undergone alterations, based on the spectroscopic features. These preliminary results proved that the availability of a spectral reference is a crucial prerequisite for an effective exploitation of spectroscopic data. On the other hand, the results also showed the great potential of using statistical algorithms in HSI data analysis for the visualization of damages, mapping alterations, and, overall, for the fast and extensive assessment of the conservation state in collections of photograms [42]. 

## 4. Discussion of Results

### 4.1. Strengths and Weaknesses of the Application of HSI to the Analysis of Photographic Negatives: Ex Post Evaluation 

The experimentation carried out with the HSI scanner on photographic negatives selected among the damaged items of the Dainelli archive pointed out weaknesses and potentialities for the use of this technique at its state-of-the-art in photography conservation. The technical feasibility of acquiring VINR hyperspectral data in transmittance on photographic negatives was proved by the demonstrator described in Section 3. The HSI transmittance scanner provided, per each 35 mm strip of six photograms, a data set including a collection of 400 narrow (about 2.8 nm bandwidth) spectral images for each examined filmstrip. 

When dealing with HSI data, different data-processing approaches can be adopted, depending on the tasks to pursue. In the following paragraphs, possible uses of the acquired data are discussed.

Overall, these preliminary results show that, at this very early stage of the research, the lack of reference spectral databases prevents a traditional spectroscopic approach to the data analysis. Nevertheless, the sequence of data images is, in principle, usable to partially retrieve the original appearance of damaged materials, as exemplified in the following paragraphs. 

#### 4.1.1. Spectroscopic Analysis Aimed at Color Recovery

In principle, a color photographic film should turn out to be a fairly simple system, having a three-layered structure, with each photo-sensitive layer containing the chemicals to selectively produce one of the three primary colors used in the tri-chromatic process [46]. However, although simple from the colorimetric point of view, such a layered system is difficult to decode from a spectroscopic point of view. In the visible region, the transmittance (or absorbance) spectra recorded feature absorption bands that turn out to be an integration of the contributions of the colored layers, applied on the support. These spectra have smooth bands, barely differentiated from each other. The discrimination of the single dye contributions could not be based on comparative analysis with known reference spectra since, in principle, they should require resorting to deconvolution mathematical methods. In addition, at present, reference spectral databases of photographic dyes and emulsions are not yet available. Therefore, the use of Vis–NIR spectra for material identification, dating or classification is still a difficult goal to reach. Nevertheless, the availability of high-resolution spectra in the visible range is expected to improve the quality of results in color recovery and digital correction procedures [47]. The HSI technique appears as the ideal candidate for this task since, if the HSI data have an acceptable spatial resolution, the spectral characteristics of individual pixels can be analyzed in detail. Generally, in order to finalize this type of intervention, it is essential to integrate the acquired data with the information reported by the manufacturers of the specific photographic films. 

Moreover, it is highly desirable measuring original films that have been impressed and developed, and are still in a good state of preservation. By doing so, it would be possible to create a specific database obtained from intact photographic films so that data from the objects under analysis can be compared to their respective reference standards. 

Although not yet usable for making spectroscopic data analyses to obtain compositional information on the examined photographic materials or their degradation products, the HSI high-resolution VNIR spectra are nevertheless suited for colorimetric analysis. As reported in [48], the HSI data are profitably usable to extract reproducible color images of the entire scene, by calculating the colorimetric coordinates per each pixel of the imaged area. This procedure is standardized according to the CIE guidelines [49]. The colorimetric values can even be converted in other colorimetric spaces, according to the applications of interest. As for other cases of art images, in the examined examples, the standard RGB (sRGB) is adopted [50].

#### 4.1.2. Image Enhancement

**Figure 4:**
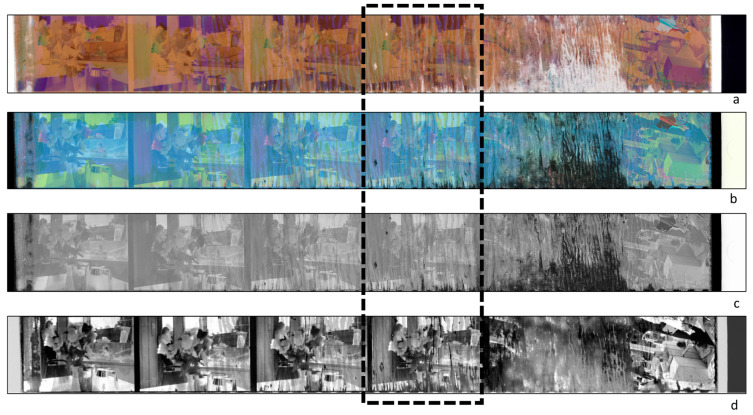
Elaborated HSI data set acquired on a damaged 35 mm film-strip negative Kodak Royal Supra 400 from the Dainelli archive: (**a**) sRGB image reconstructed from the HSI spectral data; (**b**) positive image obtained from the sRGB; (**c**) B&W image obtained by the positive image in greyscale. (**d**) PCA analysis in the 420–900 nm range: image PC3.

**Figure 5:**
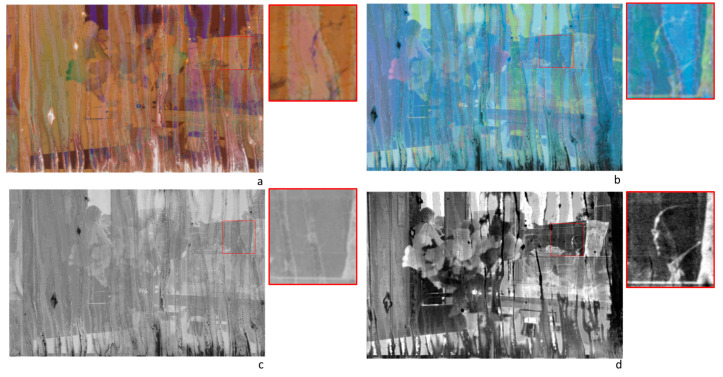
Elaborated HSI data set acquired on the fourth photogram of the film-strip negative reported in Figure 4. The fourth photogram and zoom on the selected area (red square). Comparative view of the different elaborations: (**a**) sRGB image reconstructed from the HSI spectral data; (**b**) positive color image; (**c**) B&W image obtained by the positive image in greyscale; (**d**) PCA analysis in the 420–900 nm range: PC3 image.

The sequence of the several spectral images acquired on a target surface can be processed using multivariate techniques and statistical methods. One of the tasks for which these methods can be used is image de-noising and enhancing. This enables a partial recovery of the original contents of images acquired on deteriorated supports. The suite of possible algorithms to use is wide, and an in-depth discussion of this topic is out of the scope of the present paper. In the following, a specific applicative example is reported, with the mere aim of showing the potentialities of the algorithmic approach to HSI data for image enhancing. In Figure 4, the color image sRGB of a selected sample from the Dainelli archive is reported. The examined sample was a damaged negative, 35 mm film-strip mod. Kodak Royal Supra 400, consisting of six photograms with damage at various extents. The HSI transmittance data were acquired, over the entire strip, in the 400–1000 nm range using the transmittance IFAC-CNR scanner. Starting from the HSI data set, the calibrated color image sRGB was obtained by calculating the colorimetric coordinates for each pixel (Figure 4a); the latter was converted to a negative image, thus obtaining the positive image of the scene (Figure 4b), which was, in turn, transformed as greyscale (Figure 4c). These three elaboration steps produced three digital versions of the picture impressed in negative that can be variously post-processed to highlight the visibility of the scene. However, these outputs are comparable to those of conventional digitalization processes. As a further step, the PCA algorithm was also applied to the sequence in the 420–900 nm range. Overall, the first six PC images explained 99.45% of the cumulative variance. Among the six significant PCs extracted, some of them resulted in being more informative than the conventional digital images. As an example, the PC3, which alone included 3% of the residual variance, is reported in Figure 4c. As can be noticed, some details apparently lost in the original negatives are partially retrieved in this image, and the sharpness of the image appears improved. This aspect could be ascertained by focusing inspection on the single photograms. Upon visual inspection, damage appears to be more consistent on the right side of the film, starting from the middle of the strip. The detailed view of the fourth photogram is reported in Figure 5, where the different outputs of elaborations are compared along with the magnification of a selected detail, framed as a Region of Interest (ROI). As can be observed in the sRGB image, which is the digital reproduction of the negative as it is, within the ROI area some faint contours suggest the presence of a faded face, whose details are no longer visible. Figure 5b,c show the same detail, with the face still barely visible. In Figure 5d, the PC3 shows that some traits of the visage are retrieved with particulars that seemed lost in the other images. It has to be underlined that these traits are retrieved as a result of the PCA algorithm applied to the Vis–NIR spectral sequence, and are not recoverable by means of contrast enhancement functions applied to the digital RGB. 

#### 4.1.3. Degradation Mapping 

**Figure 6:**
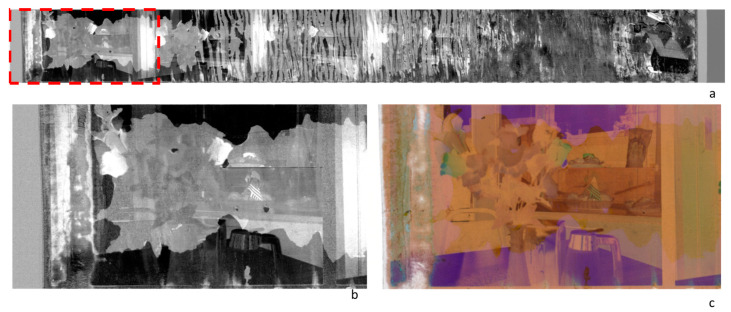
(**a**) The PC4 image obtained by the HSI data set acquired on the film-strip negative reported in Figure 4. (**b**) Detail of the first photogram in the PC4 image; (**c**) Detail of the first photogram in the sRGB image.

As further outputs, data-reduction statistical techniques, such as PCA, applied to HSI data acquired on damaged photograms are usable to map damaged areas. Indeed, the spectral decorrelation operated by the algorithm can result in images that single out the support alterations due to the damage. In Figure 6, the PC4 of the examined film strip is reported, with a magnification of the first photogram, apparently less damaged than the others on visual inspection (see also Figure 4a). It can be observed that PC4 highlights differentiated damage on the different layers of the negative and clearly singles out the contours of the altered zones.

## 5. Conclusions

A customized transmittance HSI scanner, operating in the 400–1000 nm range and designed for working on photographic negatives films and slides is illustrated. This prototype has been tested in the laboratory and also within an applicative context of interest for the field; that is, on a set of recently restored degraded photographic negatives selected from a flooded photographic archive. This pilot experimentation demonstrated the feasibility of extending the HSI imaging technique to the field of conservation of photographic heritage, and the prototype of an HSI transmittance scanner shows the technical soundness of the project. As regards the data exploitation, the elaboration performed on data sets highlighted the difficulty in using the Vis–NIR spectra directly for identification purposes, due to a lack of proper knowledge of the spectroscopic behavior of the complex layering systems of color negatives. This aspect will require further research and should address the compilation of dedicated spectral reference databases. Nevertheless, other outputs are already attainable: the data set acquired on the negatives is usable for color digital copies of the contents, where colors are accurately reproduced using the high-resolution spectral characterization pixel by pixel. In addition, the spectral data cubes are processable using multivariate algorithms, such as PCA, to obtain enhanced images that highlight the location of damage on the support, or recover details apparently lost in the damaged original. 

In addition, due to the reduced surface size, HSI can be preparatory to identify the areas in need of conservation treatment. As the discrimination between areas of total and partial loss becomes evident, the rate of lost information becomes accurate and measurable; so that, as already performed in the film restoration practice, an attempt to virtually reconstruct the image can be performed. 

## Data Availability

Restrictions apply to the availability of these data. Data was obtained within the Memoria Fotografica Project and are available from the corresponding author with the permission of Tuscany region and the photographer Daniele Dainelli.
